# Epidemiologic and clinical characteristics of severe burn patients: results of a retrospective multicenter study in China, 2011–2015

**DOI:** 10.1186/s41038-018-0118-z

**Published:** 2018-05-23

**Authors:** Hao Tian, Liangxi Wang, Weiguo Xie, Chuanan Shen, Guanghua Guo, Jiaqi Liu, Chunmao Han, Licheng Ren, Yi Liang, Yong Tang, Yuan Wang, Meifang Yin, Jiaping Zhang, Yuesheng Huang

**Affiliations:** 1 Institute of Burn Research, State Key Laboratory of Trauma, Burn and Combined Injury, Southwest Hospital, Third Military Medical University (Army Medical University), Gaotanyan Street no.29, Shapingba District, Chongqing, People’s Republic of China; 2 Institute of Burn and Plastic Surgery, the 97th Hospital of PLA, Xuzhou, , Jiangsu People’s Republic of China; 3 Department of Burns, Wuhan Third Hospital, Tongren Hospital of Wuhan University, Wuhan, Hubei People’s Republic of China; 4 The Department of Burns and Plastic Surgery, First Affiliated Hospital of People’s Liberation Army General Hospital, Beijing, People’s Republic of China; 5 The Department of Burns and Plastic Surgery, First Affiliated Hospital of Nanchang University, Nanchang, Jiangxi 330006 People’s Republic of China; 6 Institute of Burn Research Center of PLA, Xijin Hospital, Forth Military Medical University, Xi’an, Shaanxi 710032 People’s Republic of China; 7 Department of Burns and Wound Center, Second Affiliated Hospital, College of Medicine, Zhejiang University, Hang Zhou, Zhejiang, 310009 People’s Republic of China; 8 Department of Burn Reconstruction Surgery, Xiangya Hospital, Central South University, Changsha, Hunan People’s Republic of China

**Keywords:** Severe burns, Epidemiology, Multicenter

## Abstract

**Background:**

Severe burns injury is a serious pathology, leading to teratogenicity and significant mortality, and it also has a long-term social impact. The aim of this article is to describe the hospitalized population with severe burns injuries in eight burn centers in China between 2011 and 2015 and to suggest future preventive strategies.

**Methods:**

This 5-year retrospective review included all patients with severe burns in a database at eight institutions. The data collected included gender, age, month distribution, etiology, location, presence of inhalation injury, total burn surface area, depth of the burn, the length of hospitalization, and mortality. SPSS 19.0 software was used to analyze the data.

**Results:**

A total of 1126 patients were included: 803 (71.3%) male patients and 323 (28.7%) female patients. Scalds were the most common cause of burns (476, 42.27%), followed by fire (457, 40.59%). The extremities were the most frequently affected areas, followed by the trunk. The median length of hospitalization was 30 (15, 52) days. The overall mortality rate was 14.21%.

**Conclusions:**

Although medical centers have devoted intensive resources to improving the survival rates of burn patients, expenditures for prevention and education programs are minimal. Our findings suggest that more attention should be paid to the importance of prevention and the reduction of injury severity. This study may contribute to the establishment of a nationwide burn database and the elaboration of strategies to prevent severe burns injury.

## Background

Burns are devastating injuries that impair a patient’s emotional well-being and influence his or her quality of life. The World Health Organization estimates that more than 300,000 people die annually from fire-related burns worldwide [1]. Although emphasis on the prevention of burns has increased, there are still many people who have suffered burn injuries every year. In South Korea, the annual incidence of fire accidents increased from 31,372 in 2003 to 49,631 in 2008; it then decreased to 47,318 in 2012 and to 41,868 in 2013 [2]. Over 95% of victims of lethal burns live in low- and middle-income countries [3]. China currently has a population of just over 13 billion. Although > 90% of all burns are preventable, burns remain a major public health problem [4]. Severe burns can lead to extremely serious deformity, disability, or even death. The likelihood that a given individual will suffer from severe burns during his or her lifetime is 1% [5]. Patients with severe burns often need long-term hospitalization and undergo multiple surgical procedures, and some require readmission for reconstructive surgery. The impact of physical disfigurement and pain due to severe burns are far-reaching, and the psychological impairments can last a lifetime. Unfortunately, a nationwide burn database and repository, such as the American Burn Association, have not been established in China. Several studies have focused on the overall population of burn patients in different regions, including Shanghai [6], Beijing [7], and Zhejiang [8]. A multicenter study of severe burn patients for epidemiology, clinical features, and outcomes has not yet been performed in China. Here, we present the epidemiology of severe burns using a burn database that includes medical records for patients hospitalized in burn wards at eight hospitals. The data presented should contribute to the elaboration of strategies to prevent the incidence of burns, the reduction of injury severity and the development of strategies for the functional rehabilitation of burn patients.

## Methods

### Patient selection

This retrospective multicenter study included data for all severe burns patients admitted to the burn units at eight public hospitals (six academic teaching hospitals and two non-academic general hospitals) in China, from June 2011 to September 2015. Relying on the database titled “Clinical, multicentre and epidemiological case data and biological samples of severe burns database”, we included all inpatients with a diagnosis of severe burns. The criteria for entering a case into the database were as follows: (1) total burn surface area (TBSA) over 30%; (2) full-thickness burn area over 10% or full-thickness burn area over 5% for children younger than 12 years old; and (3) full-thickness burn area less than 10%, but suffered complications of shock, respiratory tract burns, or other injury-related complications. Patients with repeated admissions or incomplete data were excluded from this study. In addition, the following data were collected from electronic medical records: (1) demographic data, including ID number, age, gender, place of occurrence, admission date, and discharge date; (2) injury-related data, including the etiology of burn injuries, depth and area of the burn, injured anatomic locations, and inhalation injury; and (3) treatment situation, including the length of hospitalization and patient outcomes. The etiology of burn injuries included flame burn, scald burn, contact burn, electrical burn, chemical burn, and explosion burn. Inhalation injuries included mild, moderate, and severe inhalation injuries. Patients’ outcomes were categorized into death and cured when discharged from our center. These different outcomes were defined and evaluated based on the following criteria. If patients had died prior to discharge, the outcome was classified as “death”. If the burn wounds had healed above 95% without any secretion, the patients’ outcome was defined as “cured.”

### Quality control

All data were collected by dedicated clinical research assistants using a standardized electronic case report form. An additional researcher checked the accuracy of data input for 10% of all patients to eliminate errors due to missing or inconsistent data.

### Statistical analysis

Qualitative data were presented in the form of numbers and percentages, and the length of hospitalization was reported using medians (25th percentile, 75th percentile (Q1, Q3)). The Mann-Whitney *U* test or the Kruskal-Wallis test was conducted to compare two or more medians of categorical variables. Data were primarily input and processed using Microsoft Excel 2007. Data analysis was performed with GraphPad Prism 6 and SPSS 19.0 software. *P* < 0.05 was considered significant.

## Results

Patients who were treated in the emergency room (outpatient clinic) or did not meet the inclusion criteria of severe burn injury were not entered into the database. Ultimately, after excluding 142 patients with repeated admissions or incomplete data, 1126 patients were included in this study from January 2011 to December 2015.

### Gender and age

**Fig. 1:**
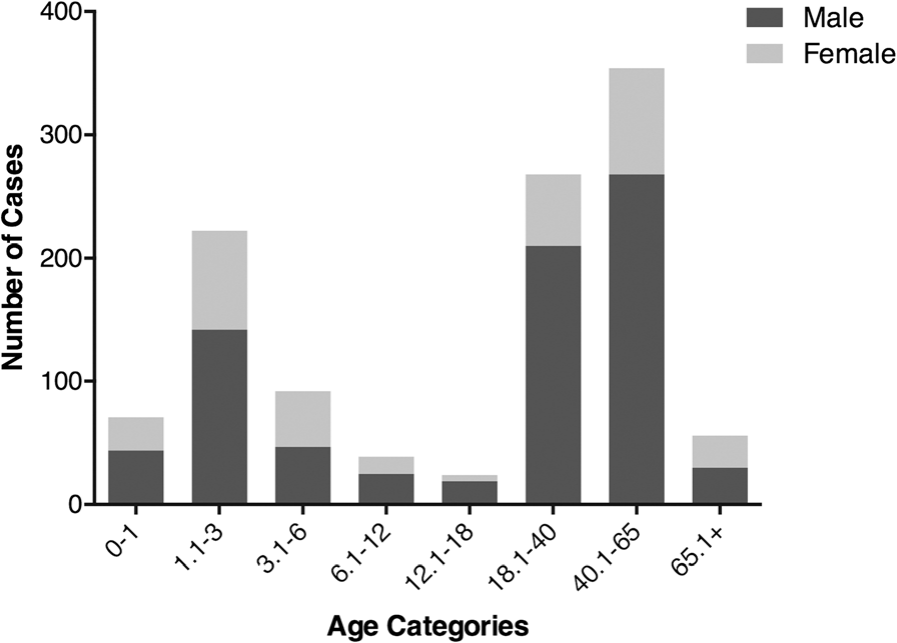
Age distribution of severe burn patients by gender group

The ratio of males to females was 2.49:1 (803 males and 323 females). The age distribution patterns of males, females, and the entire sample of inpatients appeared to be similar. The three predominant age groups of inpatients were 1.1–3 years old (222 patients, 19.72%), 18.1–40 years old (268 patients, 23.80%), and 40.1–65 years old (354 patients, 31.44%) (Fig. 1).

### Month distribution

**Fig. 2:**
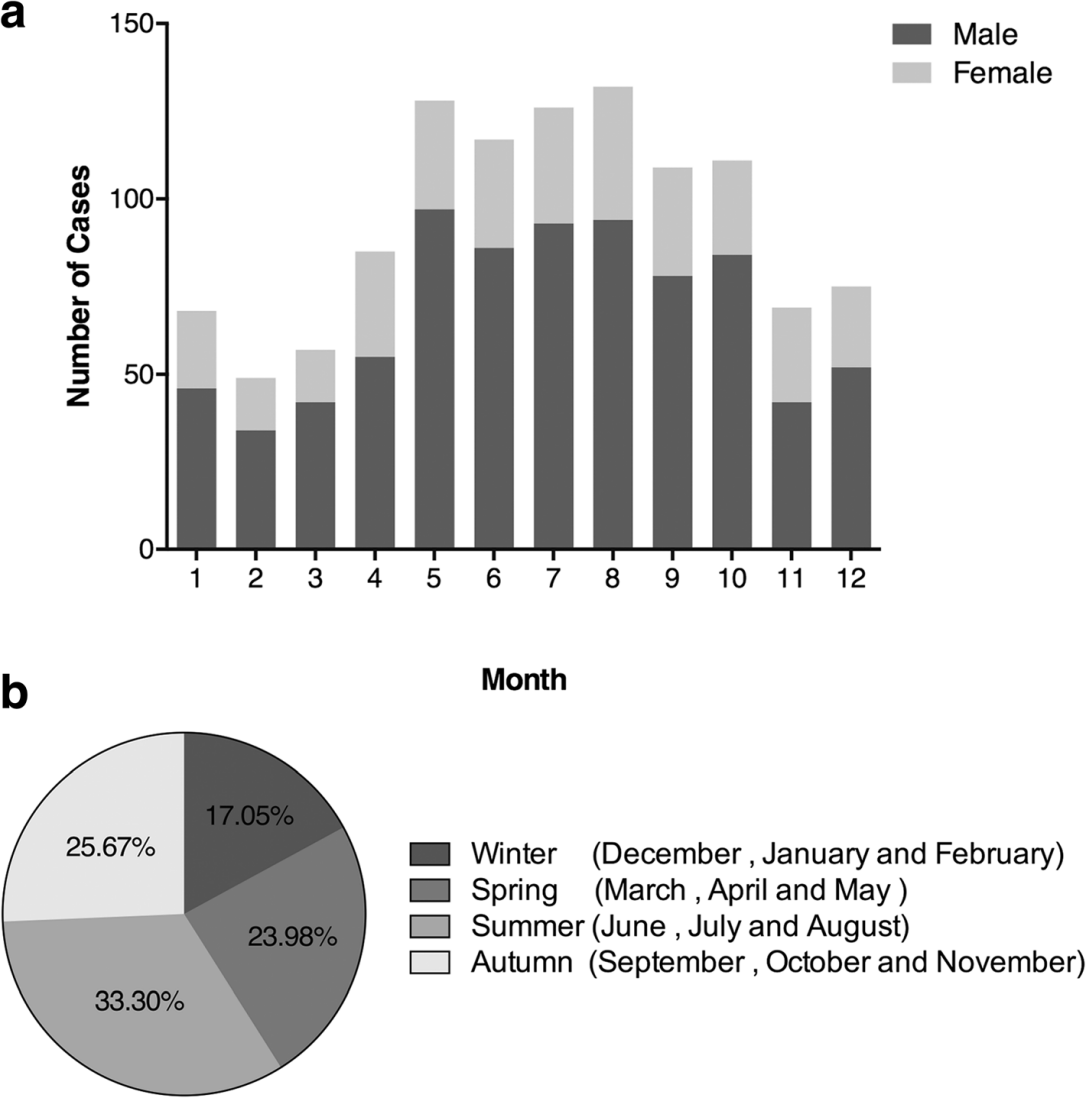
Month and season distribution of severe burn patients. **a** Number of patients being represented graphically according to month distribution in male and female groups. **b** The proportion of severe burn patients in the four seasons

Out of the entire year, burns more commonly occurred during the period from May–October than during the other months. August (132, 11.72%) had the most admissions, and February (49, 4.35%) had the fewest. Male patients’ admissions had a substantially increasing trend from February (34, 4.23%) to a peak in May (97, 12.08%); the number of admissions then showed a huge drop from October (84, 10.46%) to November (42, 5.23%). However, compared to the admission pattern of male patients, the admissions of female patients had a smooth trajectory. Female admissions increased from March (15, 4.64%) to November (38, 11.76%) and then decreased until February (15, 4.64%) (Fig. 2a). There was also a relationship between the incidence of burn and season. The summer months (June, July, and August) became the season in which individuals were the most vulnerable, with 375 (33.30%) cases. Individuals were least vulnerable to burns during the winter months (December, January, and February), with 192 (17.05%) inpatients during this period (Fig. 2b).

### Etiology and location

**Fig. 3:**
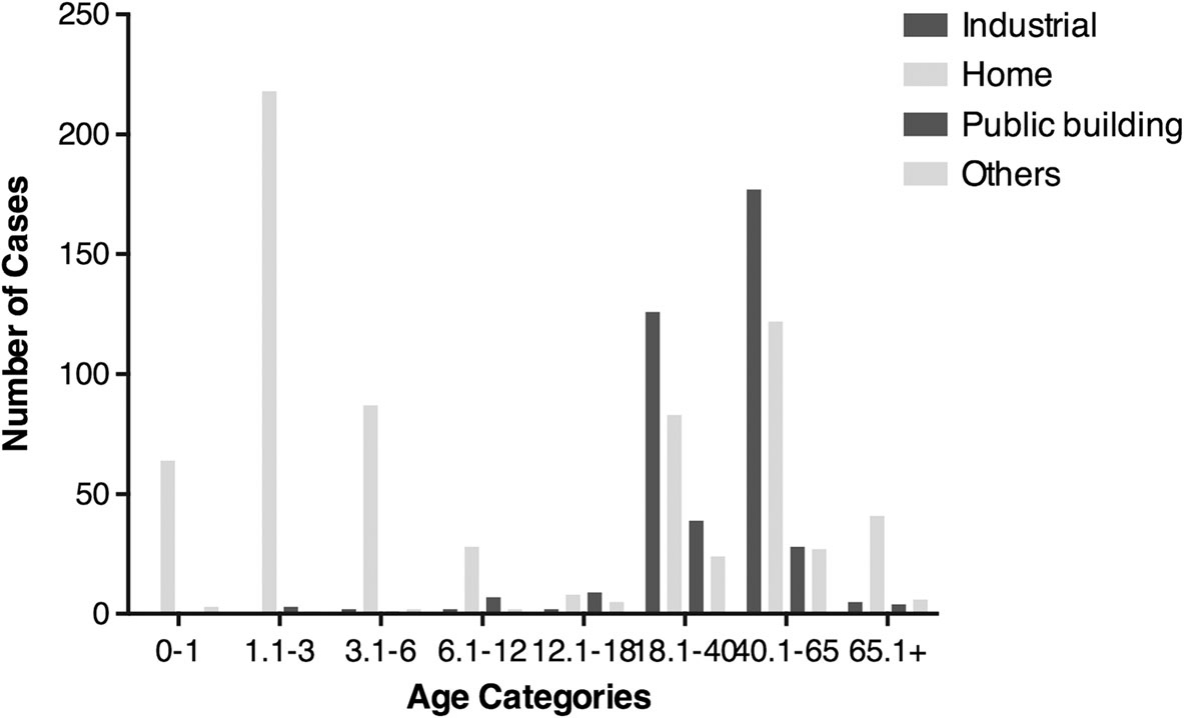
Distribution of location. Depiction of four types of location where severe burns occurred

Table 1 shows that the vast majority of burns were caused by scalds (476, 42.27%) and flame (457, 40.59%). These leading causes were followed by electrical burns (81, 7.19%), explosion burns (58, 5.15%), chemical burns (31, 2.66%), and contact burns (15, 1.33%), each of which caused significantly fewer burns than scald and flame burn. Males appeared to be more likely to suffer from electrical burns than females, while females were more likely to experience flame burns than males. The number of burns caused by flame, contact, electricity, chemicals, or explosion increased sharply when people reached working age (18.1–65 years old). In addition, burns caused by flame, electricity, chemicals, or explosion more easily occurred in the summer. The great majority of the burns were accidental and occurred at home (369, 96.85%), especially in young children. In a total of 303 adult patients (18.1–65 years old), 48.40% were work related. The reasons cited for contact, electricity, chemical, or explosion burns included failure to enforce the safety procedure and personal negligence in the workplace. Among the elderly (> 65 years of age), domestic burns (41, 73.21%) were the most common type of burn (Fig. 3).

**Table 1:** Etiology of severe burn patients by gender, age, season, and location

Etiology	Flame	Scald	Contact	Chemical	Electricity	Explosion	Others	Total
Gender, *n*(%)								
Male	320(39.85)	311(38.73)	14(1.74)	23(2.86)	77(9.59)	49(6.10)	9(1.12)	803
Female	137(42.41)	165(51.08)	1(0.31)	7(2.17)	4(1.38)	9(2.79)	0	323
M to F ratio	0.94:1	0.76:1	5.63:1	1.32:1	7.74:1	2.19:1	–	1:1
Age (years), *n*(%)								
0–1	11(15.49)	58(81.69)	0	0	1(1.41)	1(1.41)	0	71
1.1–3	9(4.05)	211(95.05)	0	1(0.45)	1(0.45)	0	0	222
3.1–6	15(16.30)	76(82.61)	0	0	1(1.09)	0	0	92
6.1–12	14(35.90)	17(43.59)	0	0	6(15.38)	2(5.13)	0	39
12.1–18	16(66.67)	1(4.17)	0	0	6(25.00)	1(4.17)	0	24
18.1–40	157(58.58)	41(15.30)	9(3.36)	8(2.99)	35(13.06)	15(5.60)	3(1.12)	268
40.1–65	188(53.11)	69(19.49)	6(1.69)	20(5.65)	29(8.19)	36(10.17)	6(1.69)	354
65.1+	47(83.93)	3(5.36)	0	1(1.79)	2(3.57)	3(5.36)	0	56
Season, *n*(%)								
Winter	90(46.88)	73(38.02)	3(1.56)	2(1.04)	16(8.33)	7(3.65)	1(0.52)	192
Spring	97(35.93)	126(46.67)	5(1.85)	7(2.59)	17(6.30)	16(5.93)	2(0.74)	270
Summer	159(42.40)	141(37.60)	3(0.80)	16(4.27)	29(7.73)	22(5.87)	5(1.33)	375
Autumn	111(38.41)	136(47.06)	4(1.38)	5(1.73)	19(6.57)	13(4.50)	1(0.35)	289
Location, *n*(%)								
Industrial	125(39.81)	72(22.93)	12(3.82)	23(7.32)	37(11.78)	38(12.10)	7(2.23)	314
Home	236(36.25)	385(59.14)	1(0.15)	4(0.61)	9(1.38)	16(2.46)	0	651
Public building	55(60.44)	7(7.69)	2(2.20)	1(1.10)	20(21.98)	4(4.40)	2(2.20)	91
Others	41(58.57)	12(17.14)	0	2(2.86)	15(21.43)	0	0	70
Total, *n*(%)	457(40.59)	476(42.27)	15(1.33)	30(2.66)	81(7.19)	58(5.15)	9(0.80)	1126

### Burn sites

**Fig. 4:**
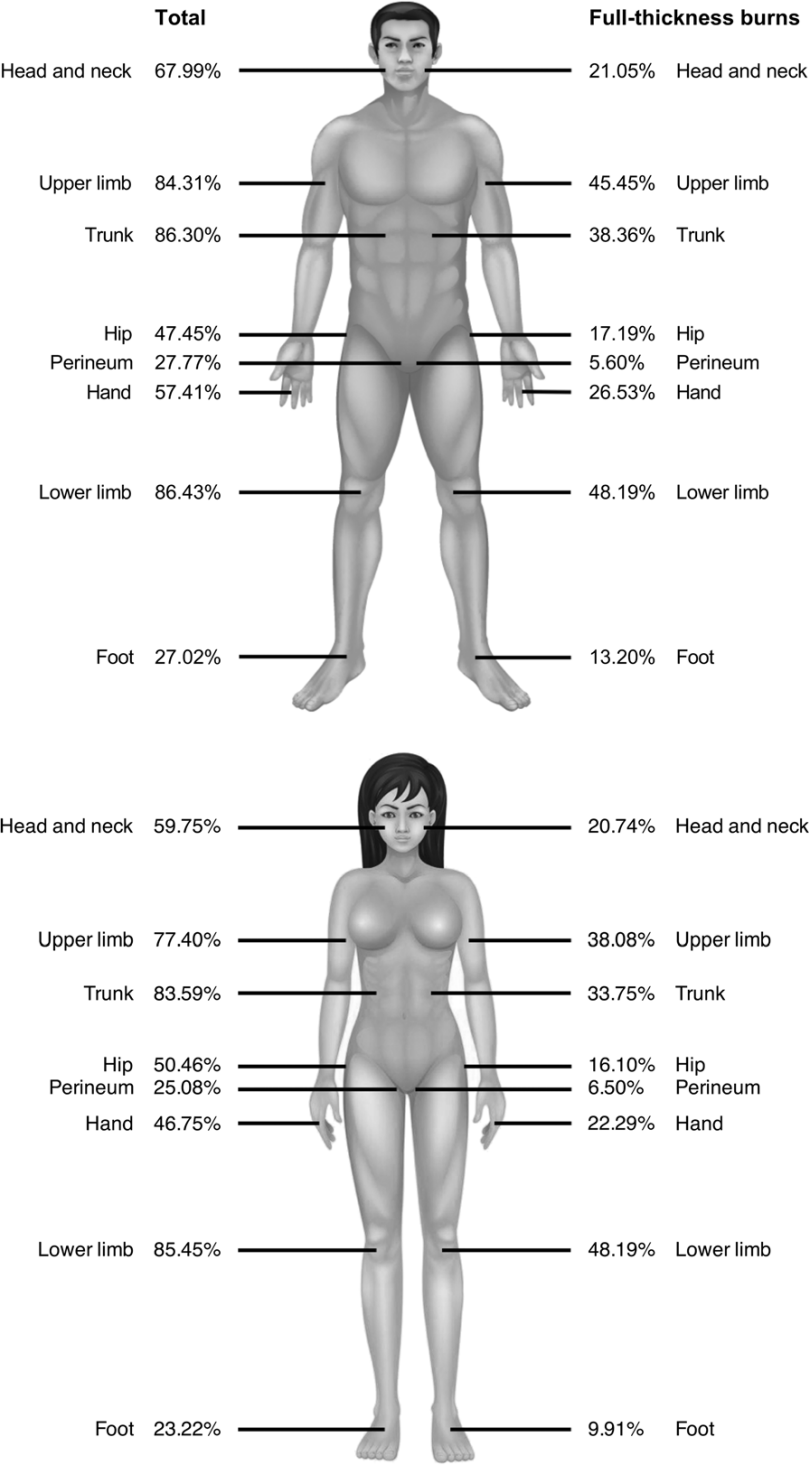
Percentage of total burns and full-thickness burns to body parts by gender group

According to collected data, we drew a burned body map, which offered a clear overview of the total burned and full-thickness burned patients. The extremities were the most frequent burned areas of lower limb, followed by the trunk. Compared with female patients, male patients tended to get burned more easily on the face and neck, upper limb, hand, and foot. On the basis of the amplitude between the total burn and the full-thickness burn sites, the head and neck were protected well compared to the extremities of all patients (Fig. 4).

### TBSA

**Fig. 5:**
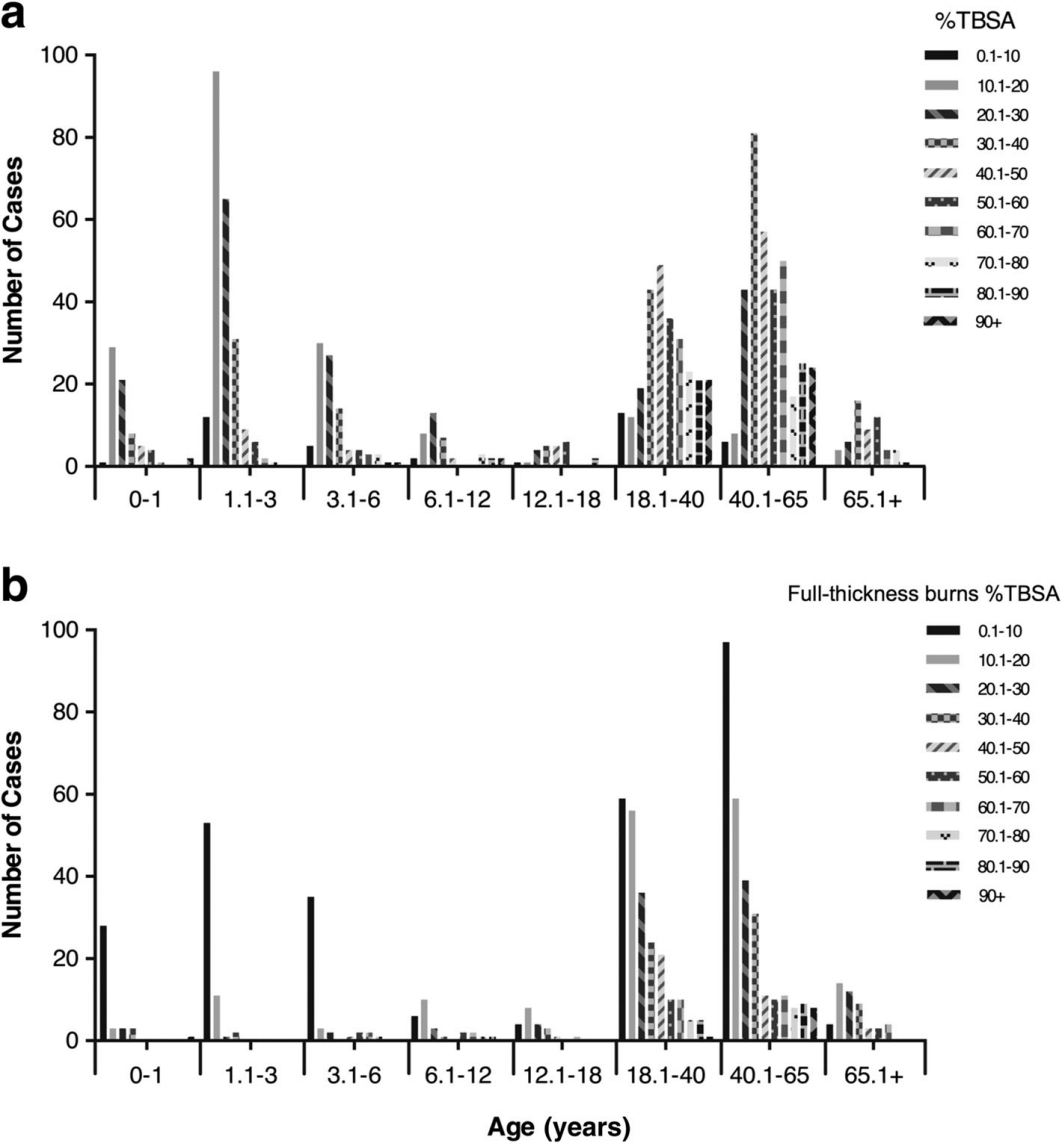
Distribution of % total body surface area (TBSA) and full-thickness burns %TBSA by age. **a** Number of patients according to the percentage of TBSA involved with burns by age group. **b** Number of patients according to the percentage of TBSA involved with full-thickness burns by age group

**Fig. 6:**
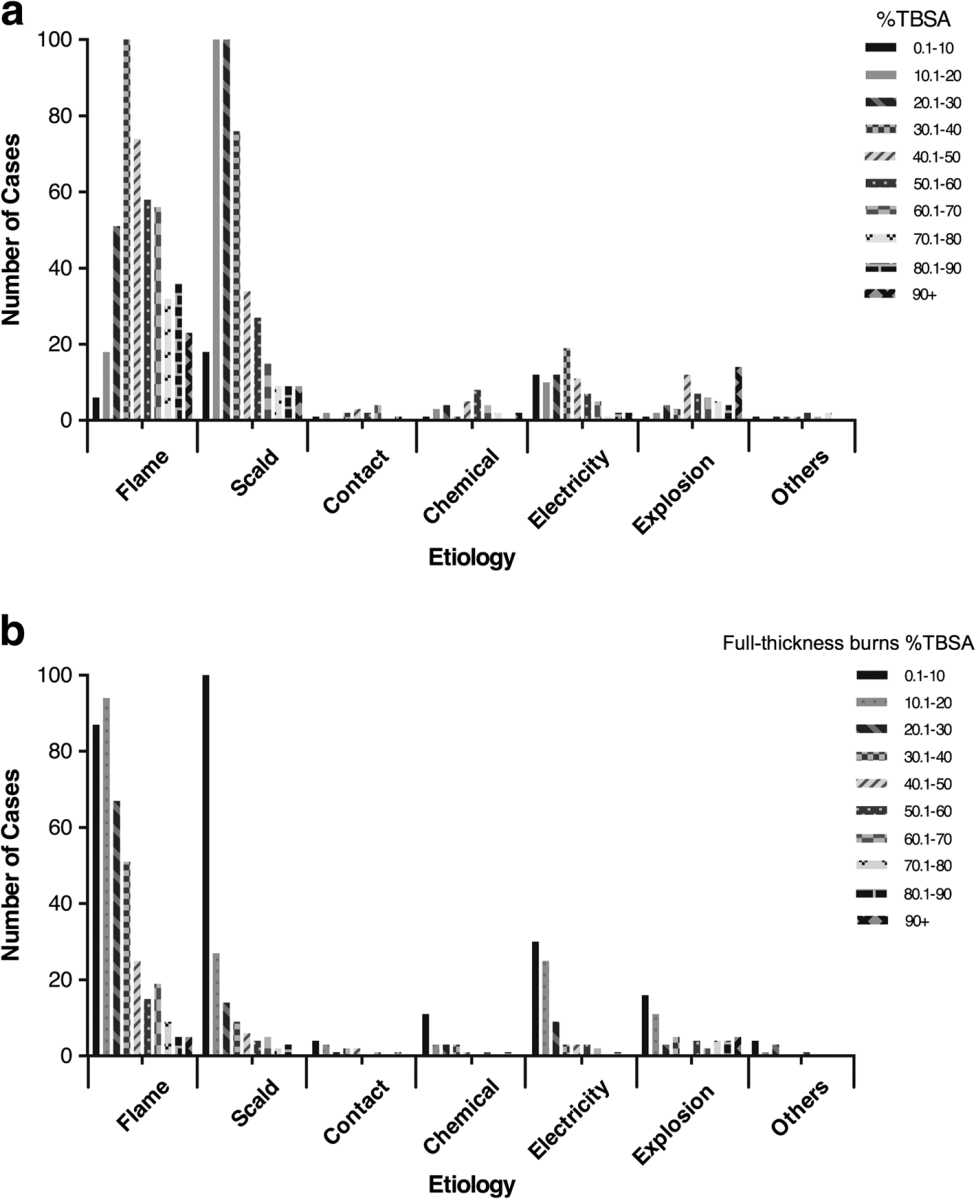
Distribution of % total body surface area (TBSA) and full-thickness burns %TBSA by etiology. **a** Number of patients according to the percentage of TBSA involved with burns by etiology group. **b** Number of patients according to the percentage of TBSA involved with full-thickness burns by etiology group

TBSA of the full-thickness burns in age had a similar trend with the overall TBSA (Fig. 5). Most young patients (0–6 years old) suffered a burn with a TBSA of less than 30%, while most older patients (45+ years old) had a TBSA of greater than 30.1%. Among scald burns, most TBSA ranged from 10.1–30%. For flame burns, most TBSA were above 30.1% (Fig. 6a). Among all the patients, 757 (67.23%) severely burned patients had full-thickness burns. In contrast to the causes in overall burn patients, full-thickness burns were most commonly caused by flame (377, 49.8%), followed by scald (204, 26.9%) (Fig. 6b).

### The length of hospitalization

**Fig. 7:**
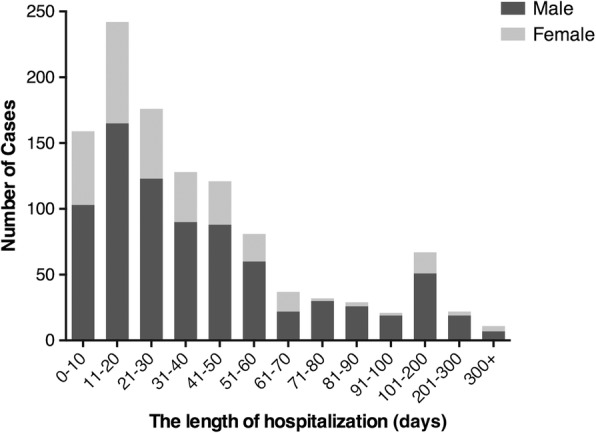
Number of severe burns patients represented graphically according to the length of hospitalization by gender group

The overall median length of hospitalization was 30 (15, 52) days, ranging from 1 to 576 days. Table 2 shows the results of median length of hospitalization associated with epidemiological features, and Fig. 7 illustrates the detailed distribution of the length of hospitalization by gender. A total of 73.4% of the patients (826) were hospitalized < 50 days, and among them, the 11- to 20-day group had the largest numbers (242) of patients. The median length of hospitalization of male patients was 31 (16, 55) days, and the median length of hospitalization of female patients was 26 (14, 45) days. There is a significant difference in the length of hospitalization by gender (*z* = − 3.176, *p* < 0.05).

**Table 2:** The length of hospitalization and mortality rate by gender, age, season, etiology, inhalation injury, %total body surface area (TBSA) and full-thickness burns %TBSA

	The length of hospitalization		Mortality		
	Median (Q1, Q3), days	Statistic, *P* value	Died cases, *n*	Total cases, *n*	Mortality rate, *n*%
Gender		114,009, < 0.05			
Male	31(16, 55)		105	803	13.08
Female	26(14, 45)		55	323	17.03
Age		194.5, < 0.001			
0–1	26(14, 37)		8	71	11.27
1.1–3	18(11, 28)		19	222	8.56
3.1–6	25(13, 41)		6	92	6.52
6.1–12	26(21, 34)		7	39	17.95
12.1–18	50(28, 65)		3	24	12.50
18.1–40	47(25, 79)		25	268	9.33
40.1–65	38(20, 64)		54	354	15.25
65.1 +	7(1, 40)		38	56	67.86
Season		4.7, 0.196			
Winter	27(12, 48)		39	192	20.31
Spring	29(17, 52)		36	270	13.33
Summer	29(15, 55)		47	375	12.53
Autumn	31(16, 54)		38	289	13.15
Etiology		75.8, < 0.001			
Flame	38(17, 61)		83	457	18.16
Scald	23(14, 37)		44	460	9.57
Contact	54(21, 97)		2	15	13.33
Electrical	42(19, 81)		8	81	9.88
Chemical	41(19, 69)		5	30	16.67
Explosion	39(19, 74)		17	58	29.31
Others	27(28, 170)		1	15	6.67
Inhalation injury		25.3, < 0.001			
Mild	39(23, 70)		18	139	12.95
Moderate	46(14, 81)		39	146	26.71
Severe	38(11, 95)		19	44	43.18
Total	40(15, 47)		76	329	23.10
TBSA		110.4, < 0.001			
0.1–10	19(12, 28)		2	40	5.00
10.1–20	18(11, 32)		14	188	7.45
20.1–30	26(15, 41)		22	198	11.11
30.1–40	32(16, 52)		23	198	11.62
40.1–50	37(21, 64)		14	140	10.00
50.1–60	38(20, 58)		16	111	14.41
60.1–70	45(25, 87)		10	91	10.99
70.1–80	47(23, 78)		14	51	27.45
80.1–90	55(19, 99)		20	59	33.90
90+	33(11, 79)		25	50	50.00
TBSA 3rd degree		123.4, < 0.001			
None	19(13, 31)		21	369	5.69
0.1–10	34(20, 52)		22	286	7.69
10.1–20	45(23, 65)		24	164	14.63
20.1–30	44(22, 79)		17	100	17.00
30.1–40	45(12, 90)		19	73	26.03
40.1–50	42(12, 97)		13	37	35.14
50.1–60	54(17, 122)		7	27	25.93
60.1–70	34(6, 98)		14	30	46.67
70.1–80	37(23, 63)		7	15	46.67
80.1–90	22(14, 90)		9	15	60.00
90+	11(4, 32)		7	10	70.00
Total	30(15, 52)		160	1126	14.21

*Q1* 25th percentile, *Q3* 75th percentile

### Mortality

Among the 1126 admitted patients, 160 (14.21%) died, including 105 (13.08%) males and 55 (17.03%) females. Patients aged 65.1+ years had the highest mortality (38, 67.86%) in all age groups, while children 3.1–6 years old had the least (6, 6.52%). The highest mortality rate was observed in winter (39, 20.31%). Explosion burns have been associated with a higher mortality rate (17, 29.31%), followed by burns due to flame (83, 18.16%). Inhalation injuries were observed in 329 cases (29.2%). The number of patients with mild, moderate, and severe inhalation injuries was 139, 146, and 44, respectively. Of those with inhalation injury, the mortality rate was 34.35%, significantly higher than the 22.56% average mortality. A higher mortality tends to emerge when the TBSA and the TBSA of full-thickness burns increase (Table 2).

## Discussion

### Gender and age

Children account for a large proportion of the population with severe burns injury [9]. In addition, children younger than 5 years old account for 50 to 80% of all childhood burns [10, 11]. The children between 1 and 6 years old (314, 27.89%) were the most likely to be severely burned compared to children under 1 year of age (71, 6.31%) or between 6 and 18 years old (63, 5.60%). As children grow up, they receive less protection from parents and lack adequate self-protection abilities, which results in the first summit in Fig. 1. In most studies, an overall predominance of males among burn injury patients (55 to 75%) has been described [12, 13]. More men than women work in poor and dangerous environments and suffer larger burned skin areas. The increased risk for severe burns among males aged 18–65 years may reflect inadequate protection measures at the workplace. Children under age 4 and the elderly have a higher chance of complications and death from severe burns because their skin tends to be thinner than that of patients in other age groups. Accordingly, children and the elderly need more protection from family members, and workplaces should offer more protection to adults, especially males.

### Month distribution

The seasonal (rather than yearly) variation in burn admission seems more statistically valuable for the study. Different populations and injury sites, causes, and the like have a connection with the environmental temperature, as has been previously reported [14]. The same phenomena in other studies, a peak in summer months, were described in our study [15, 16]. People are more easily sunburned during the summer because they wear less clothing and thus have less protection against the sun. However, summer has the lowest mortality rate, while winter has the highest. Most likely, in winter, the cold limits the range of people’s activities, making electric blankets, fireplaces, and flammable fuels more easily available and fatal in use. Additionally, a long period of direct contact between the skin and hot-water bottles or a crack in the bottles leads to severe burns.

### Etiology and location

In contrast to previously published epidemiologic findings, we found that scald burns, rather than fire burns, were the most common cause of severe burns [17, 18]. However, findings from our study are the same as those from the studies from Catalonia and Diyarbakir [19, 20]. In pediatric populations, scald burns account for 81% of all burns requiring hospitalization. Young children were more likely to be injured by boiling water, hot soup, or hot milk. Different types of burns are distinctly related to the environmental temperature, dietary habits, bathing habits, and clothes. For the location of burns, our study showed similar demographic characteristics, with most chemical, electrical, and explosion burns occurring during the performance of work-related activities. However, fire burns and hydrothermal scald burns typically occurred in the home [15]. In the workplace, males are usually more active and exposed to higher environmental risks.

### Burn sites

As for the sites of burns, the face and neck, trunk, and extremities were the most easily burned parts of patients of all age group, which is comparable with the findings of a study in Shanghai, China [6]. For the first time, we put the total burned sites and the sites of full-thickness burns into a burned body map, and the sites that are more vulnerable to severe burns can be reflected by the reduced amplitude. Approximately 60% of patients were burned on the head and neck, but less than one-third of patients (20%) experienced full-thickness burns, possibly because individuals subconsciously protect their head and other important parts of their body when confronted with sudden injury. The protection of the head and perineum is admittedly necessary; however, we should pay particular attention to burns on the extremities. Those burned areas not only cause cicatrix and deformities but also bring about functional losses, thus affecting patients’ quality of life.

### TBSA

Burns of < 20% TBSA do not represent a large proportion of severe burns. This trend reflects the strict admission criteria at many institutions [7, 21]. Large-area burns mainly occur in working-age adults. The most common etiology among adults was flame burn; the most common etiology among children was scald burn. Compared to scald burn, flame-sustained burns were associated with increased TBSA, as determined by the injury mechanism. It also explained that full-thickness burns were most commonly caused by flames. The full-thickness burns are considered one of the main risk factors of death. The TBSA of full-thickness burns is often the key factor affecting the final outcome. The importance of developing etiology-based prevention and treatment strategies is crucial to decreasing the mortality and morbidity caused by severe burn injuries.

### The length of hospitalization

Patients with serious injuries were typically kept until their wounds had nearly healed. Our department also provides rehabilitation for burn patients, resulting in prolonged hospital stays. Accordingly, the length of hospitalization may have been longer than the time required for wound treatment. This result suggests that new measures may be necessary to shorten the average length of hospitalization. We recommend shortening the interval between operations and increasing the size of the area treated during a single operation. The final declining trend of the average length of hospitalization with the increasing age and TBSA may be due to increased mortality and thus reduced the length of hospitalization.

### Mortality

Mortality rate was closely related to the proportion of burn patients with severe burns. The mortality rate for most hospitalized patients with severe burns ranges from 1.4 to 18% (maximum 34%) [22]. Although other variables are also related to a higher mortality, older age, inhalation injury, and increasing TBSA are the three major risk factors for mortality in our study, which is consistent with reports from other studies.

The elderly are prone to burns because of age-related decreases in mobility, cognition, and judgment. Twenty six (8%) female burn patients aged over 65 while 30 (3.7%) male burn patients aged over 65, which may explain the higher mortality of females than males. Owing to their weak physical basis and pre-burn diseases, the elderly have higher mortality rates and worse prognosis relative to other age groups. The aging of China’s population is becoming increasingly serious, and the elderly population with severe burns remains a challenging problem. There is a clear correlation trend between inhalation injury and mortality. It is well known that the presence of inhalation injury significantly increases mortality among burn patients [23, 24]. Overall, inhalation injury occurred in 29.2% of all hospitalized patients with severe burns injuries but in 47.5% of all deceased patients. In addition, more attention should be paid to comorbidities at the time of admission. Among the patients included in this study, the most common comorbidity was respiratory disorder (e.g., inhalation injury). Acute upper airway obstruction occurs in 20 to 33% of hospitalized burn patients with inhalation injury and is a major hazard because of the risk for rapid progression from mild pharyngeal edema to complete upper airway obstruction [25]. The prognosis is made worse by respiratory damage, which is the second-most common cause of death after the injury. However, a patient with very severe burns may die, even in the absence of inhalation injury. Currently, the treatment of inhalation injury includes antibiotics, airway control, and mechanical ventilation. More evidence-based individualized protocols should be developed to treat burns in patients with a high risk of death. Generally, a comparison of correlations of mortality rate, TBSA, and deep burn area shows that the mortality rate increased significantly with the increase of the TBSA and the depth of the burn. Interestingly, the 20.1–70% TBSA groups have similar mortality rates. It is possible that many patients with burns with TBSA under 30% received treatment and were therefore not included in this study. There are several limitations to this study. The database “Clinical, multicentre and epidemiological case data and biological samples of severe burns database” had been established before the execution of this study but still has room for improvement. Many data points were excluded due to clinical deficiencies, which may have resulted in deviations related to outcomes.

## Conclusions

Medical centers devote intensive resources to improving the survival rates among burn patients. Further efforts to improve the quality of life for burn survivors should eventually have a very beneficial effect in this patient population. Analyzing the epidemiological information of burns serves to prepare each patient and provide the best service to relieve their pain rather than to distinguish the treatment of burn patients. Based on these epidemiological characteristics, some specific suggestions can be made. Therefore, some programs should take action through, for instance, strengthening health promotion, child supervision, and labor protection, all of which may reduce the incidence of burns. The establishment of a multicenter database indicates a future development trend to finally build a nationwide burn database and repository in China.

## References

[1] Smolle C, Cambiaso-Daniel J, Forbes AA, Wurzer P, Hundeshagen G, Branski LK (2017). Recent trends in burn epidemiology worldwide: a systematic review. Burns.

[2] Han TH, Kim JH, Yang MS, Han KW, Han SH, Jung JA (2005). A retrospective analysis of 19,157 burns patients: 18-year experience from Hallym Burn Center in Seoul, Korea. Burns.

[3] Elberg JJ, Schroder HA, Glent-Madsen L, Hall KV (1987). Burns: epidemiology and the effect of a prevention programme. Burns Incl Therm Inj.

[4] Ahn CS, Maitz PK (2012). The true cost of burn. Burns.

[5] Organization WH. A WHO plan for burn prevention and care. Bull World Health Organ. 2008;87:802-3. 10.2471/BLT.08.059733 PMC2755313 19876549

[6] Fan X, Ma B, Zeng D, Fang X, Li H, Xiao S (2017). Burns in a major burns center in East China from 2005 to 2014: incidence and outcome. Burns.

[7] Cheng W, Yan-hua R, Fang-gang N, Wei-li D, Guo-an Z (2012). Epidemiology of 1974 burn patients at a major burn center in Beijing: a nine-year study. J Burn Care Res.

[8] Ye C, Wang X, Zhang Y, Ni L, Jiang R, Liu L (2016). Ten-year epidemiology of chemical burns in western Zhejiang Province, China. Burns.

[9] Dedovic Z, Brychta P, Koupilova I, Suchanek I (1996). Epidemiology of childhood burns at the Burn Centre in Brno, Czech Republic. Burns.

[10] Anlatici R, Ozerdem OR, Dalay C, Kesiktas E, Acarturk S, Seydaoglu G (2002). A retrospective analysis of 1083 Turkish patients with serious burns. Burns.

[11] Koller J, Orsag M, Ondriasova E, Graffinger I, Bukovcan P (1994). Analysis of 1119 burn injuries treated at the Bratislava Burn Department during a five-year period. Acta Chir Plast.

[12] Dhopte A, Tiwari VK, Patel P, Bamal R (2017). Epidemiology of pediatric burns and future prevention strategies-a study of 475 patients from a high-volume burn center in North India. Burns Trauma.

[13] Dhopte A, Bamal R, Tiwari VK (2017). A prospective analysis of risk factors for pediatric burn mortality at a tertiary burn center in North India. Burns Trauma..

[14] Dokter J, Vloemans AF, Beerthuizen GI, van der Vlies CH, Boxma H, Breederveld R (2014). Epidemiology and trends in severe burns in the Netherlands. Burns.

[15] Liu Y, Chen JJ, Crook N, Yu R, Xu XW, Cen Y (2013). Epidemiologic investigation of burns in the elderly in Sichuan Province. Burns.

[16] Morita S, Higami S, Yamagiwa T, Iizuka S, Nakagawa Y, Yamamoto I (2010). Characteristics of elderly Japanese patients with severe burns. Burns.

[17] Schiefer JL, Perbix W, Grigutsch D, Zinser M, Demir E, Fuchs PC (2016). Etiology, incidence and gender-specific patterns of severe burns in a German Burn Center––insights of 25 years. Burns.

[18] Seo DK, Kym D, Yim H, Yang HT, Cho YS, Kim JH (2015). Epidemiological trends and risk factors in major burns patients in South Korea: a 10-year experience. Burns.

[19] Barret JP, Gomez P, Solano I, Gonzalez-Dorrego M, Crisol FJ (1999). Epidemiology and mortality of adult burns in Catalonia. Burns.

[20] Aldemir M, Kara IH, Girgin S, Guloglu C (2005). Factors affecting mortality and epidemiological data in patients hospitalised with burns in Diyarbakir, Turkey. S Afr J Surg.

[21] Tripathee S, Basnet SJ (2017). Epidemiology of burn injuries in Nepal: a systemic review. Burns Trauma..

[22] Brusselaers N, Monstrey S, Vogelaers D, Hoste E, Blot S (2010). Severe burn injury in Europe: a systematic review of the incidence, etiology, morbidity, and mortality. Crit Care.

[23] Darling GE, Keresteci MA, Ibanez D, Pugash RA, Peters WJ, Neligan PC (1996). Pulmonary complications in inhalation injuries with associated cutaneous burn. J Trauma.

[24] Osler T, Glance LG, Hosmer DW (2010). Simplified estimates of the probability of death after burn injuries: extending and updating the Baux score. J Trauma.

[25] Kobayashi K, Ikeda H, Higuchi R, Nozaki M, Yamamoto Y, Urabe M (2005). Epidemiological and outcome characteristics of major burns in Tokyo. Burns.

